# Rapid Operationalization of COVID-19 Immunization Clinics With Medical and Physician Assistant Students Serving as Vaccinators

**DOI:** 10.1016/j.focus.2024.100199

**Published:** 2024-02-02

**Authors:** Robert D. Bradshaw, Cynthia C. Romero, Jovanna A. Tracz, Lydia Lukomski, Julie L. Stoner, Shambhawi Thakur, Michael A. Wilson

**Affiliations:** 1 Eastern Virginia Medical School, Norfolk, Virginia; 2 Department of Family and Community Medicine, Eastern Virginia Medical School, Norfolk, Virginia; 3 Department of Occupational Health, Eastern Virginia Medical School, Norfolk, Virginia; 4 M. Foscue Brock Institute for Community and Global Health, Eastern Virginia Medical School, Norfolk, Virginia; 5 School of Health Professions, Eastern Virginia Medical School, Norfolk, Virginia

**Keywords:** Medical education, COVID-19 vaccination, public health, infectious disease, preventive medicine

## Abstract

**Introduction:**

In anticipation of institutional and community-wide COVID-19 immunization clinics, an educational program for the administration of COVID-19 vaccines was developed, collaborating with the Virginia Department of Health to train doctor of medicine and physician assistant students to serve as vaccinators. Faculty and students also worked with state legislatures to propose and enact a bill that would enable such students to vaccinate patients.

**Methods:**

Between January 2021 and August 2022, 263 student volunteers completed 3,685 person-hours, administering 48,279 doses. On the basis of community need, the majority of vaccines were administered at mass vaccination clinics in Chesapeake (47%) and Norfolk (22%) in Virginia. One year after the first COVID-19 immunization clinic, the authors surveyed students who assisted with clinics, utilizing a Likert scale and free-text responses to elicit feedback about the training and volunteer experience.

**Results:**

Volunteers ranked the vaccination clinics among the top third of established volunteer experiences offered at Eastern Virginia Medical School, and 75% of respondents believed that this training should be a permanent part of their program curriculum.

**Conclusions:**

This paper presents a strategy for preparing students to serve as vaccinators during public health emergencies and show how other institutions of medical education can prepare for and engage student participation in vaccination campaigns and emergent health initiatives.

## HIGHLIGHTS

• This paper presents a strategy for preparing students to serve as vaccinators during public health emergencies
• Faculty and students worked with legislatures to propose and enact bills to enable doctor of medicine and physician assistant students to vaccinate patients.
• A total of 263 student volunteers administered 48,279 COVID-19 vaccinations between January 2021 and August 2022.
• The majority of vaccines were administered at mass vaccination clinics on the basis of community need.
• Students ranked vaccination clinics among the top third of established volunteer experiences.

## INTRODUCTION

After the outpouring of volunteer healthcare worker response after the 9/11 terrorist disaster in New York City, public health officials recognized a need for better preparation, training, certification, and organization of volunteers.^1^ Consequently, health departments in the U.S. developed the Medical Reserve Corps, which includes both active and retired nurses, physicians, emergency medical technicians, and other health professionals, as well as nonmedical volunteers to assist in public health administrative and support roles. Medical Reserve Corps across the U.S., including the authors’ state of Virginia, were mobilized effectively during the 2009 H1N1 influenza pandemic to staff and implement mass immunization clinics, and in 1 survey of health departments, 85% of respondents listed mass immunization among the top 5 most important emergency preparedness and response activities.^2^ Nursing students have been utilized for general disaster response in the past, including immunizations, after Hurricane Harvey in Texas and during an on-campus influenza outbreak.^3^^,^^4^ In addition, pharmacy professional associations have advocated to be included in these activities.^5^

In December 2019, several cases of pneumonia of unknown etiology occurred in Wuhan, Hubei province, People's Republic of China.^6^ In January 2020, this virus was identified as severe acute respiratory syndrome coronavirus 2 (SARS-CoV-2), the cause of coronavirus disease 2019 (COVID-19), and by March 2020, the WHO declared COVID-19 a global pandemic.^7^ Owing to the international spread and the large burden of morbidity and mortality of COVID-19 on healthcare systems worldwide, a multinational effort was made to identify therapeutics and immunizations that protect against this disease. According to WHO, initially, there were up to 180 vaccine candidates and dozens in clinical trials.^8^

When the Pfizer-BioNTech COVID-19 vaccine, BNT162b2, received Emergency Use Authorization from the U.S. Food and Drug Administration (FDA) in December 2020, healthcare institutions across the nation rapidly prepared vaccination clinics. The initial BNT162b2 vaccine challenged vaccination facilities, for example, with required storage of vaccines at ultralow temperatures (−80°C to −60°C) and strict cold chain requirements, vaccine dilution and mixing protocols, and injection within 6 hours after mixing.^9^ Strict protocols complicated initial vaccination rollout and necessitated adequate education regarding cold-chain processes to prevent vaccine wastage.^10^^,^^11^ As a result, the need for additional vaccinators led some U.S. schools of health professions to begin training students as vaccinators.^12^, ^13^, ^14^, ^15^

Early in the pandemic, students at Harvard Medical School published a proposal for the role of students in assisting during the pandemic. The clinical support roles described did not anticipate the use of medical students in mass immunization clinics as vaccinators.^16^ However, the American Medical Association “encourages physicians, residents, and medical students to participate in disaster response activities through organized groups, such as the Medical Reserve Corps…and not as spontaneous volunteers.”^17^ A 2021 study by Rebman et al.^18^ found that when shortages in qualified vaccine-administering personnel are anticipated during public health emergencies, “other health science students, such as medical students…could also be recruited to aid in mass vaccination at Points of Dispensing.”

This article describes an initiative undertaken to train and utilize health professions students in the doctor of medicine (MD) and physician assistant (PA) programs at Eastern Virginia Medical School (EVMS) to augment mass immunization efforts during the COVID-19 pandemic. The authors (1) describe a sample curriculum and reference model legislation that other schools of health professions may adopt to enhance disaster response and (2) identify optimal elements of training and clinic logistics to be prioritized in mass immunization clinics, with the goal that sharing this educational program may serve as a model for future public health initiatives.

## METHODS

**Figure 1:**
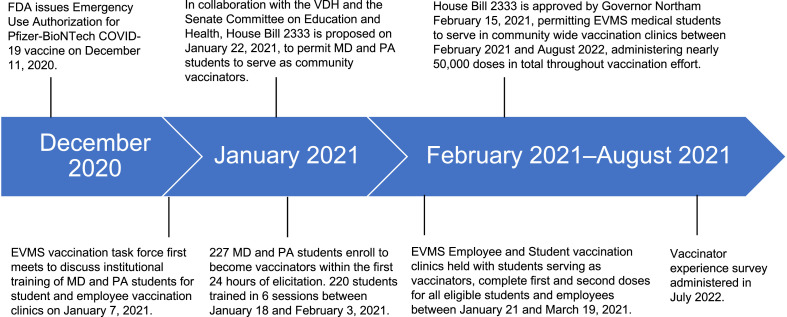
Timeline of COVID-19 vaccination events starting from the first emergence of COVID-19 in December 2019 and ending with participant survey administration in January 2022. EVMS, Eastern Virginia Medical School; FDA, Food and Drug Administration; MD, doctor of medicine; PA, physician assistant; VDH, Virginia Department of Health.

Faculty at EVMS, including the Director of Occupational Health (former Virginia Department of Health [VDH] Health District Director), and the Director of the M. Foscue Brock Institute for Community and Global Health (former Virginia Health Commissioner) recognized that widespread immunization of employees, students, and the community would require a concerted effort beyond the resources of the existing public health system on the basis of experiences gained during the H1N1 pandemic.^19^ Between the first vaccination strategy meeting on January 15, 2021 and the first EVMS employee and student vaccination clinic on January 21, 2021 (Figure 1), a team consisting of the Director of Occupational Health; the Director of the M. Foscue Brock Institute for Community and Global Health; a medical student serving as the student vaccination clinic and volunteer coordinator; the EVMS Director of Finance, Human Resources, and Facilities; the Nursing Clinical Quality Coordinator; and a Professor of Family and Community Medicine collaborated to acquire, store, and reconstitute Pfizer-BioNTech vaccines, schedule patients, and recruit and train volunteers for MD and PA student-run vaccination clinics. Presented here is an overview of this process.

### Legislative Considerations

Before vaccinating the greater Hampton Roads community in collaboration with VDH, faculty had to work with state legislatures to update applicable regulations. At the onset of the pandemic, Virginia only allowed nursing and pharmacy technician students to administer immunizations under faculty supervision during public health emergencies. Thus, a new regulation (House Bill 2333) was proposed to enable MD and PA students and other similar professionals in training to vaccinate recipients with appropriate liability protections.^20^ This bill was passed in February 2021.^21^

### Recruitment of Students From the Eastern Virginia Medical School Doctor of Medicine and Physician Assistant Programs as COVID-19 Vaccinators

Students’ interest in being trained to administer the Pfizer-BioNTech vaccine was gauged using an online form (Google Surveys) distributed via e-mail to all MD and PA classes. Two hundred twenty-seven students from the EVMS MD and PA programs registered to assist in the vaccination effort within the first 24 hours of the training announcement. This suggested significant student interest in assisting with community vaccination efforts, thus an application process was used to select a medical student to serve as the COVID-19 student vaccination clinic and volunteer coordinator. This student was tasked with serving as a liaison between administrators and students, leading the initiative on behalf of the students, and working with both public health officials and EVMS faculty to train and schedule students for vaccination events. As this role evolved, an assistant coordinator role was also created.

After completing the required vaccination training (Section III), MD and PA students were added to an email server to register for upcoming vaccination events through an online scheduling platform (SignUpGenius).^22^ SignUpGenius software also enabled the tabulation of total student volunteer hours over time.

Students first assisted as vaccinators during internal EVMS employee and student immunization clinics in January 2021. Upon passage of House Bill 2333^21^ in February of 2021, students were able to partner with the VDH and several regional healthcare organizations to host community immunization events, which ranged from roughly 200 to 1,500 immunizations per day. Students were supervised using a ratio of 1 supervising physician to 12 trained MD or PA students. In addition to vaccinator roles undertaken by the MD and PA students, other students enrolled in programs within the School of Health Professions, including the Pathologist Assistant and Master of Surgical Assisting Program, volunteered in nonvaccinator roles such as registering patients and transporting prefilled syringes from the vaccine reconstitution tables to vaccinator stations. These roles were essential in facilitating clinic flow and increasing patient throughput.

### Development of Vaccination Training and Workshops

With feedback from the VDH, the Director of Occupational Health and the student vaccination clinic and volunteer coordinator created and optimized an educational program for handling and intramuscular (IM) injection of mRNA COVID-19 vaccines, with an emphasis on the BNT162b2 vaccine. The vaccinator training curriculum included (1) an online module with a postmodule quiz that MD and PA students were required to complete with a score of ≥80% and (2) in-person training with simulated injection techniques using model arms. The presentation in both online and in-person training components included the following topics:

1. Background regarding the novel coronavirus pandemic, development of the Pfizer-BioNTech (and later, Moderna) mRNA vaccines, results of Phase III COVID-19 mRNA vaccination trials, and the meaning of FDA Emergency Use Authorization.
2. Manufacturer-specific training for the specific vaccine(s) students would administer; predominantly Pfizer-BioNTech mRNA vaccine; later, manufacturer information regarding mRNA-1273 and Ad26COVS1 was added.
3. Identification of potential adverse vaccine reactions, including patient anaphylaxis, and proper use of an EpiPen.
4. Safe use of multidose vials.
5. Proper administration of IM vaccines using the Z-track method.^23^

Students were also referred to the VDH online training certification for clinical volunteers. Finally, during the vaccination clinic, the student was required to observe 3 injections and perform 3 injections under the supervision of VDH or EVMS faculty, consistent with VDH requirements for all clinicians administering vaccines. The students were given constructive feedback during each of the 3 injections to ensure safe and proper technique for vaccine administration. After 3 successful injections, the students were deemed competent to vaccinate under indirect supervision (at a ratio of 12 students to 1 physician) and were then able to vaccinate independently.

### Eastern Virginia Medical School COVID-19 Community Event Considerations

Beginning in February 2021, faculty at the M. Foscue Brock Institute for Community and Global Health also worked with vaccination coordinators at VDH and hospital and pharmacy locations throughout the Hampton Roads area to provide EVMS MD and PA student vaccinators the support to assist with greater community vaccination efforts. Off-campus vaccination clinics took place at community centers, including temples, churches, schools, and public housing, to target medically underserved populations that were disproportionately affected by the pandemic.^24^ Faculty tracked student support and regularly reported to the dean of EVMS regarding total immunizations given and hours volunteered. Volunteer hours counted toward the American Association of Medical Colleges–required community-engaged learning (CEL) activities. Faculty limited events to no more than 2 per week and monitored scheduling to avoid conflicts with academic schedules.

### Vaccinator Experience Survey

In January 2022, one year after the first COVID-19 immunization clinic was held at EVMS, collaborators administered a survey through the Qualtrics platform^25^ to student volunteers who assisted with vaccination clinics between January and May 2021. The survey was approved by the EVMS IRB (RB#22-05-XX-0107) and utilized both the Likert Scale and free-text responses to elicit feedback regarding the training and volunteer process as well as factors contributing to successful mass immunization events. Responses were voluntary and anonymous and were not linked to grades or academic evaluation. The Dillman technique for survey recruitment was utilized, including an initial email invitation and 2 weekly reminders: an initial reminder after the first week of administration and another a week later to maximize response rate.^26^

### Statistical Analysis

A sample Z-test analysis was performed using the R package Basic Statistics and Data Analysis software (R Foundation) with an assumed normal distribution with central tendency. A *p*<0.05 was considered significant. Qualitative analysis was utilized to identify, code, and categorize participant responses. Major themes were identified with participant quotes from free-text responses.

## RESULTS

### Volunteer Data

During the program, 303 students were trained, and 263 participated in institutional and community vaccination. From January 21, 2021 to August 16, 2022, 263 student volunteers clocked a total of 3,685 person-hours toward community vaccination efforts, administering 48,279 doses in total at EVMS and community clinics. Clinics included those arranged by EVMS, Hague Pharmacy, Peoples Pharmacy, Chesapeake Regional Medical Center, Sentara, and the VDH. Student volunteer participation peaked early in the vaccination effort: 26,417 and 14,875 doses were given in the months of March and April 2021, respectively. Most vaccines were administered at mass vaccination clinics in Chesapeake, Virginia (47%), followed by Norfolk, Virginia (22%), on the basis of community need. There were no recorded patient complications during vaccination clinics at which students served as vaccinators, aside from occasional routinely managed vasovagal responses.

### Vaccinator Experience Survey

Half the respondents were first-year medical students (50%) with no prior experience administering IM injections (83%) (Table 1). Forty-eight percent of students had participated in both student and community vaccination events; 25.4% of students participated in community vaccination events only. For 63% of students, vaccination training was their first in-person clinical training experience since the start of the pandemic. The most common reason cited for volunteering was to “serve the community in an essential effort” (*p*=0.02), followed by “to have fun” (i.e., study break, change of scenery, volunteer with friend group) (*p*=0.04) (Table 1).

**Table 1:** Survey Participant Demographics

Variable	Survey responses, *n* (%)	
Education level		
PA-1	5 (8.1%)	
PA-2	1 (1.6%)	
MS-1	31 (50.0%)	
MS-2	20 (32.3%)	
MS-3	4 (6.5%)	
MS-4	0 (0.0%)	
Training and clinical experience		
Training only	5 (7.5%)	
Training and student clinical event	8 (11.9%)	
Training and community clinical events	17 (25.4%)	
Training and both student and community events	32 (47.8%)	
No training	5 (7.5%)	
First in-person training experience since start of the COVID-19 pandemic		
Yes	38 (63.3%)	
No	22 (36.7%)	
Previous experience giving intramuscular injections		
Yes, during clinical rotations	0 (0.0%)	
Yes, as a nurse, EMT, or other medical professional	10 (16.7%)	
None	50 (83.3%)	
	*n*=67	

	Average Ranking (of 7^a^)	*p*-value
Reasons for participating		
To work with my fellow EVMS students and faculty	4.21±1.85	0.4168
To serve the community in this essential effort	1.86±1.06	**0.0162**
To gain a new clinical skill early in my medical career	3.45±1.61	0.2912
To join a historic collaborative public health response helping save lives	3.40±1.83	0.2743
To personally take actions to help our society return to normalcy	3.74±1.72	0.3974
To build my resume for postmedical school/PA school applications	5.55±1.29	0.0606
To have fun (i.e., study break, change of scenery, volunteer with friend group)	5.79±1.41	**0.0367**
	*n*=42	

*Note:* Boldface indicates statistical significance (*p*<0.05).

a 1 indicates ranked as most important to the student. EMT, emergency medical technician; EVMS, Eastern Virginia Medical School; PA, physician assistant.

Students generally were satisfied with the content of both in-person and online trainings: ≥80% of students supported the inclusion of background information regarding the pandemic, vaccination clinical trials, emergency use authorization, vaccine cold chain, and dilution processes as well as video instructions about injection technique and use of an EpiPen (Table 2). Students unanimously (100%, *p*=0.003) favored the ability to ask questions directly to instructors during the in-person training. Ninety-three percent of students agreed with the requirement of direct observation and supervision of 3 IM injections by a physician in the clinical setting prior to administration of vaccines independently.

**Table 2:** Student Satisfaction With Vaccination Training and Clinic Scheduling

Variable *n*=67	Strongly/somewhat disagree, n (%)	Neither agree nor disagree, n (%)	Strongly/somewhat agree, n (%)	*p*-value
Background information on COVID-19 infection	1 (1.8%)	1 (1.8%)	54 (96.4%)	**0.0074**
Video on deltoid intramuscular injection	1 (1.8%)	0 (0.0%)	55 (98.2%)	**0.0029**
Video on treatment of anaphylaxis with Epi-Pen	5 (8.9%)	6 (10.7%)	45 (80.4%)	**0.0291**
Hands-on simulation training with syringes	1 (1.8%)	2 (3.6%)	52 (92.9%)	**0.0048**
Information on clinical trial of Pfizer-BioNTech vaccine	2 (3.6%)	8 (14.3%)	45 (80.4%)	**0.0291**
Information on cold chain precautions with vaccine	7 (12.5%)	3 (5.4%)	46 (82.1%)	**0.0421**
Information on dilution and premixing vaccine	6 (10.7%)	6 (10.7%)	44 (78.6%)	0.0525
Information on other FDA emergency use authorization vaccines	4 (7.1%)	2 (3.6%)	50 (89.3%)	**0.0140**
Ability to ask questions directly to instructors during the training	0 (0.0%)	0 (0.0%)	56 (100.0%)	**0.0032**
Direct observation and supervision of 3 intramuscular injections	2 (3.6%) *n*=56	2 (3.6%)	52 (92.9%)	**0.0074**
The scheduling software was easy to use and understand	2 (3.9%)	7 (13.5%)	43 (82.7%)	**0.0204**
Notice for clinics gave adequate time for sign-up	9 (17.3%)	4 (7.7%)	39 (75.0%)	0.0772
I preferred clinics held on weekends	4 (7.7%)	14 (26.9%)	34 (65.4%)	0.0817
I preferred clinics on school days if no class conflicts	8 (15.4%)	18 (34.6%)	26 (50.0%)	0.1898
I preferred clinics in locations closer to EVMS	0 (0.0%)	8 (15.4%)	44 (84.6%)	**0.0157**
I preferred large venue clinics (e.g., churches)	2 (3.9%)	20 (38.5%)	30 (57.7%)	0.0729

*Note:* Bolded values denote statistical significance (*p*<0.05).

EVMS, Eastern Virginia Medical School; FDA, Food and Drug Administration.

Eighty-three percent of volunteers were satisfied with the scheduling software (*p*=0.02). Volunteers generally preferred clinics located closer to the EVMS campus (85%, *p=*0.02) (Table 2). Students ranked the following 3 elements as most important to clinic workflow: use of prefilled syringes (average ranking 1.63/10), patients being already checked in and prescreened for vaccine contraindications (average ranking 2.57/10), and patients having relevant questions answered before they approached the vaccination stations (average ranking 2.57/10) (Table 3).

**Table 3:** Most Important Aspects of the Clinical Setting in Mass Vaccination

Variable *n*=49	Average ranking (of 10^a^)	*p*-value
Use of prefilled syringes	1.63±1.04	**0.0049**
Having patients already checked in and prescreened for contraindications	2.57±1.60	**0.0254**
Having an administrative assistant in support of the vaccinator	5.59±2.54	0.4761
Having patients get relevant questions answered while waiting	4.84±2.17	0.3300
Use of vanish point syringes	6.33±2.52	0.2900
Having separate access for patients with mobility issues	7.22±2.02	0.1258
Having a separate, monitored vaccine waiting area	7.47±1.65	0.0945
Being able to exchange needle length on the basis of body habitus and weight	7.41±2.03	0.1015
Having vaccine runners replace syringes and other items when stations run low	5.12±2.34	0.4000
Use of a flag system to denote when vaccine station is open for new patients	6.82±2.8	0.1894

*Note:* Bolded values denote statistical significance.

a 1 indicates ranked as most important to the student

Overall, volunteers ranked vaccination clinics among the top third of established volunteer experiences offered at EVMS, and 75% believed that this training should be a permanent part of their program curriculum. Qualitative review of free-text responses revealed the following 2 themes: community interaction and valuable skill-building experience.

## DISCUSSION

MD and PA students may serve as a supplemental workforce in vaccine administration when healthcare staff are experiencing workplace burden during a global pandemic. Students can reduce this burden while positively contributing to public health, as demonstrated by the 48,279 doses safely administered by 263 EVMS student volunteers between January 2021 and August 2022. High student-to-supervisor ratios and no recorded adverse events other than an occasional vasovagal reaction during vaccination clinics support entrusting properly trained students with the clinical activities discussed in this paper. Furthermore, students were highly motivated to serve the local community in an essential effort, exemplified by survey responses, including, “There's no other activity that I've done in which I felt like I was making a bigger impact than the vaccine clinics. I got to help my community return back to normal. It's an experience I won't ever forget,” “I believe that student vaccinating provided more real benefit to the community/patients (and more useful experience for students) than a lot of other CELs [Community Engaged Learning] currently in operation,” and “...my most effective and involved CEL experience.”

Vaccinator survey results also revealed the importance of (1) comprehensive background information in training, (2) in-person interaction for hands-on practice, and (3) initial direct observation by supervising clinicians in preparing students with adequate skill and confidence in the administration of new vaccines. Standardized training with practice on model arms allowed all students to acquire this skill, and most volunteer respondents supported the formal implementation of this training into the program curriculum.

Collaboration with clinic staff for the optimization of clinic flow and ease of use for student vaccinators was essential in volunteer satisfaction and continued service. Although students preferred clinics closer to the Norfolk EVMS campus, the majority (78%) of vaccine doses were administered in surrounding cities (Chesapeake, Portsmouth, Virginia Beach, Suffolk, and Hampton), demonstrating student willingness to travel to communities in need. Student volunteer participation peaked within the first 3 months of the vaccination effort, as expected with a decrease in need and clinic visit frequency after May 2021. This was followed by an increase in volunteer participation in April and May 2022, correlating with the FDA's approval of a second booster for certain populations announced in March 2022.^27^ The continuing need for the skills discussed in this paper supports medical student action in serving local community health efforts.

### Limitations

This experience was limited by the region served owing to the location of EVMS. Vaccines were administered in urban and suburban settings, with minimal rural vaccine distribution. The evaluation survey was potentially subject to both recruitment and response bias. The student population was composed more of students earlier in their professional education, including mostly first- and second-year medical students, because clinical rotations limited participation by students later in their studies. Furthermore, the response rate was limited to 47 of over 200 eligible survey respondents. Subsequently, this may have resulted in more students responding who viewed the experience positively.

## CONCLUSIONS

The program presented in this paper utilized student volunteers to supplement vaccination efforts during the COVID-19 pandemic, demonstrating that it may be replicated for other public health emergencies. The initiative benefited greatly from the state-level public health experience of authors RDB and CCR, which expedited the passage of enabling legislation. Although the authors recognize that situations vary in different states, this experience suggests that knowledge in advance regarding state and federal guidelines facilitates constructive action.

Student fulfillment derived from this work in conjunction with the development of clinical skills early in medical education suggests the potential for continued implementation. This framework included open communication across the state health department, medical school, and student leadership. Volunteer competency and satisfaction with training required comprehensive background information, hands-on skills, practice with clinicians, and supervision of skill acquisition by clinical staff. This model promotes a meaningful experience for medical students to advance their skills while safely and effectively contributing to public health. Medical and public health institutions, perhaps using designated emergency preparedness staff, may wish to review present legislation to ensure that students can quickly mobilize to enhance internal and community public health emergency preparedness and response.

## CRediT authorship contribution statement

**Robert D. Bradshaw:** Conceptualization, Methodology, Supervision, Writing – original draft. **Cynthia C. Romero:** Conceptualization, Data curation, Methodology, Supervision, Writing – review & editing. **Jovanna A. Tracz:** Conceptualization, Methodology, Writing – original draft. **Lydia Lukomski:** Conceptualization, Methodology, Writing – original draft. **Julie L. Stoner:** Conceptualization, Data curation, Methodology, Supervision, Writing – review & editing. **Shambhawi Thakur:** Software, Visualization, Writing – original draft. **Michael A. Wilson:** Software, Visualization, Writing – original draft.
